# A Comprehensive Strategy for Screening for Xenotransplantation-Relevant Viruses in a Second Isolated Population of Göttingen Minipigs

**DOI:** 10.3390/v12010038

**Published:** 2019-12-29

**Authors:** Luise Krüger, Yannick Kristiansen, Emelie Reuber, Lars Möller, Michael Laue, Christian Reimer, Joachim Denner

**Affiliations:** 1 Robert Koch Institute, HIV and Other Retroviruses, 13353 Berlin, Germany; KruegerL@rki.de (L.K.); KristiansenY@rki.de (Y.K.); ReuberE@rki.de (E.R.); 2 Robert Koch Institute, Centre for Biological Threats and Special Pathogens ZBS 4: Advanced Light and Electron Microscopy, 13353 Berlin, Germany; MoellerL@rki.de (L.M.); LaueM@rki.de (M.L.); 3 Department of Animal Sciences, University of Goettingen, Animal Breeding and Genetics Group, Albrecht-Thaer-Weg 3, 37075 Göttingen, Germany; christian.reimer@agr.uni-goettingen.de; 4 Center for Integrated Breeding Research, University of Goettingen, Albrecht-Thaer-Weg 3, 37075 Göttingen, Germany; 5 Robert Koch Institute, Robert Koch Fellow, 13353 Berlin, Germany

**Keywords:** porcine viruses, xenotransplantation, porcine endogenous retroviruses (PERV), porcine cytomegalovirus (PCMV), hepatitis E virus (HEV), porcine lymphotropic herpesvirus (PLHV), porcine circovirus (PCV)

## Abstract

Xenotransplantation using pig tissues and organs is under development in order to alleviate the increasing shortage of human transplants. Since xenotransplantation may be associated with the transmission of porcine microorganisms to the human recipient, the donor pigs should be carefully analyzed, especially for the presence of potentially zoonotic viruses. Göttingen Minipigs (GöMP) are potential donors of islet cells for the treatment of diabetes. Despite the fact that all animals produced at Ellegaard Göttingen Minipigs A/S carry porcine endogenous retroviruses (PERVs) in their genome and that very few animals were infected with porcine cytomegalovirus (PCMV), hepatitis E virus (HEV) and porcine lymphotropic herpesvirus (PLHV), no transmission of these viruses was observed in a preclinical trial transplanting GöMP islet cells into cynomolgus monkeys. Using a new comprehensive strategy, we then analyzed an isolated subpopulation of Göttingen Minipigs which remained at the University of Göttingen. We concentrated on 11 xenotransplantation-relevant viruses and combined co-incubation assays with susceptible human target cells and molecular biological methods to evaluate the risk posed by PERV. All animals in Göttingen carry PERV-A, PERV-B, and PERV-C in their genome but they are not infected with PCMV, PLHV and HEV. The difference may be explained by selection of negative animals and/or de novo infection. The PERV copy number was established using ddPCR (93 copies) and a human-tropic PERV-A/C was found released from PBMCs of one animal with a high expression of PERV-C.

## 1. Introduction

Göttingen Minipigs (GöMP) are well characterized and broadly used in biomedical research [1,2,3]. GöMP are the result of crossbreeding Minnesota Minipigs, Vietnamese potbelly pigs and German landrace pigs at the University of Göttingen, Germany [4]. Later the commercial production of GöMP was performed at Ellegaard Göttingen Minipigs A/S (Denmark) in a full-barrier specified pathogen-free (SPF) facility. At Ellegaard the animals are screened twice a year for numerous microorganisms including 27 bacteria, 16 viruses, three fungi and four parasites [3]. Among the porcine viruses, PERVs pose a special risk for xenotransplantation, since all pigs carry proviruses in their genome [5]. PERV-A and PERV-B are capable of infecting human cells and are present in the genome of all pigs, whereas PERV-C infects only pig cells and is present in the genome of many, but not all pigs. When we analyzed GöMP from Ellegaard for the prevalence and expression of PERVs, we found that all animals carry PERV-A, PERV-B and PERV-C [6]. However, recombinant PERV-A/Cs were not found in five investigated Ellegaard GöMP. PERV-A/C has been found in some organs of other pig breeds carrying PERV-A and PERV-C, they are the product of recombination and de novo integration into somatic pig cells of living pigs and never have been found in the germ line of pigs [7]. PERV-A/C are characterized by higher replication rates compared with their parental viruses [8] and further passaging on human cells increased their virus titer due to duplications of repeats in the LTR [9]. Using droplet digital PCR, 82 copies of PERV were found in GöMP from Ellegaard [10]. The expression of PERV, analyzed as full-length mRNA using reverse transcriptase (RT)-PCR with specific primers, was found moderate to low, the expression increased after stimulation of PBMCs with the mitogen phytohemagglutinin (PHA) [6]. However, release of human-tropic PERV, able to infect human 293 cells was not observed in these experiments [6]. Together with the screening performed at Ellegaard, 49 groups of pathogens (more than 88 individual microorganisms) were analyzed and found absent in GöMP [11]. Using highly sensitive RT-PCR and immunological methods we found hepatitis E virus (HEV), porcine cytomegalovirus (PCMV) and porcine lymphotropic herpesviruses (PLHV) in a very small number of GöMP from Ellegaard [11,12]. HEV was shown to be transmitted via the placenta, making elimination more difficult [11]. At present HEV is the only known zoonotic porcine virus able to infect humans and to induce disease [13,14]. PCMV was found to reduce significantly the survival time of pig xenotransplants in non-human primates and therefore may also pose a risk when pig organs will be transplanted into humans (for review see [15]). Despite the presence of low amounts of PCMV, PLHV and HEV in very few GöMPs from Ellegaard, no transmission of these viruses was observed in a preclinical trial transplanting islet cells from these pigs into cynomolgus monkeys [16].

Here we analyze a subpopulation of the GöMP founder population which remained and was bred isolated from the Ellegaard GöMP at the University of Göttingen. Interestingly, in this population all animals were free of PCMV and HEV and therefore in these points different from the GöMP at Ellegaard. The analysis was based on a new screening strategy and was concentrating on xenotransplantation-relevant viruses, first of all PERVs.

## 2. Materials and Methods

### 2.1. Pig Holding Including Absence of Vaccination and Food Additives

The Relliehausen breeding colony is housed in a conventional indoor stable with specific pathogen free status, located on the research farm of the University of Göttingen, near Dassel, Germany. The facility is functionally separated into mating, farrowing, weaning and general maintenance units. Admission to the facility requires passing of a hygienic lock and use of in-house clothing. Animals are fed a conventional fibre rich pelleted diet and straw from the adjunct fields is administered for enrichment reasons. In case of disease, animals are treated according to veterinary standards, but no regular vaccination scheme is employed. The colony is under a regular health monitoring which involves testing for *Actinobacillus pleuropneumoniae* ApxII-toxin, pseudorabies (porcine herpesvirus 1, PHV1), classical swine fever, Mycoplasma hyponeumoniae, porcine reproductive and respiratory syndrome virus (PRRSV, Betaarterivirus suid), Sarcoptes scabiei var. suis, salmonella, Pasteurella multocida and several endoparasites. Animal keepers who also work with other pigs must maintain a two-day quarantine before re-entering the facility. In principle, no animals are brought or returned into the facility.

### 2.2. Blood and Isolation of PBMCs

Eleven animals of the Relliehausen breeding colony of different age and different gender were tested repeatedly (Table 1). EDTA blood was taken vom the Vena jugularis (permission number 33.9-42502-05-19A391, Niedersächsisches Landesamt für Verbraucherschutz und Lebensmittelsicherheit) and shipped cooled at 4 °C. PBMCs were isolated using a Lymphocyte Separation Medium 1077 (PromoCell, Heidelberg, Germany) gradient.

### 2.3. General Screening Strategy

**Figure 1:**
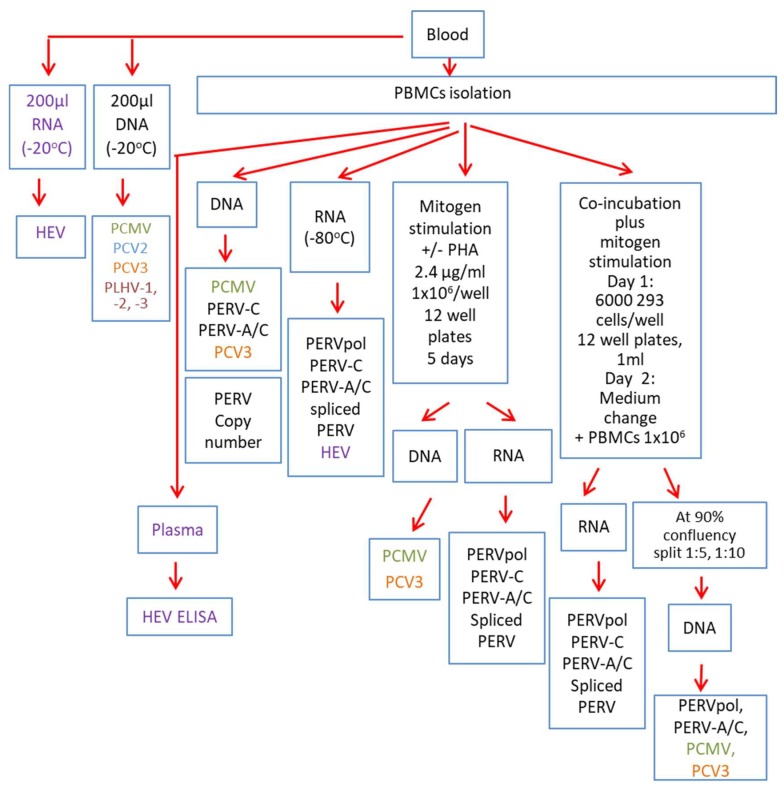
Schematic presentation of the screening strategy.

Using blood and isolated PBMCs, a broad virological analysis of the GöMP was performed (Figure 1).

### 2.4. DNA and RNA Isolation

DNA was extracted from sera, blood or PBMCs using different DNA extraction kits: DNeasy Blood and Tissue kit (Qiagen GmbH, Hilden, Germany), and NucleoSpin Virus (Macherey-Nagel, Düren, Germany). DNA was quantifiedand the 260 nm/280 nm ratio was determined using a NanoDrop ND-1000 (Thermo Fisher Scientific Inc., Worcester, MA, USA). RNA was extracted from blood or cells using RNeasy Mini kit (Qiagen GmbH, Hilden, Germany) following the manufacturer’s instructions.

### 2.5. PCR, RT-PCR and Real-Time PCR

**Figure 2:**
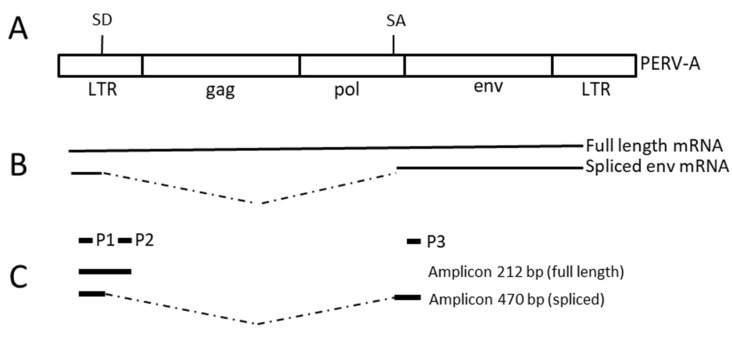
(**A**), Genomic organisation of PERV proviruses, (**B**), transcription of the full-length mRNA (coding for the Gag proteins and the enzymes) and the spliced mRNA (coding for the envelope proteins only), (**C**), localisation of the primers P1, P2 and P3 to amplify the spliced mRNA by RT-PCR and length of the amplicons [23] (Table 2). SD, splice donor; SA, splice acceptor; LTR, long terminal repeat; gag, pol, env, sequences coding for the Gag, Pol and Env proteins; bp, base pairs.

PCR methods or reverse transcriptase PCR (RT-PCR) methods, respectively, (Table 2) were performed to screen for HEV [11,17], PCMV [12,18], PCV2 [19], PCV3 [20], PLHV (-1, -2, -3) [11] as described. Using a primer pair in the pol region of PERV (PERVpol) all PERV types were detected [21], using specific primers for the envelope regions, PERV-C and PERV-A/C were detected [7,22]. To detect expression of PERV, primers specific for PERVpol were used which detect the full-length mRNA. The full-length mRNA encodes Gag and Pol proteins. Furthermore, using specific primers, spliced mRNA was detected encoding the envelope proteins (Env) (Figure 2, Table 2) [23]. Finally, using specific primer in the env region, the expression of PERV-C was analysed. In real-time PCRs porcine GAPDH was used for normalisation (Table 2).

### 2.6. Mitogen Stimulation and Co-Culture

Based on previous investigations showing that mitogen stimulation increases the expression of PERV in pig PBMCs up to the release of infectious virus particles [6,24,25,26], PBMCs from the GöMP were treated with 2.4 µ/mL, in some cases 9.6 µg/mL phytohemagglutinin-L (PHA-L, M5030, Biochrom GmBH, Berlin, Germany) and the expression of PERV was analyzed at the mRNA level and by testing release of human-tropic virus. In a co-incubation assay with human embryonic kidney 293 T cells, which are highly susceptible to an PERV infection due to the absence of intracellular restriction factors among them APOBEC3G [27], the ability of released virus particles to infect human cells was analysed (Figure 1). The co-cultures were repeatedly splitted to remove the pig PBMCs and 293 T cells were screened repeatedly for PERV infection by PCR. To make sure that there were no remaining pig cells a PCR was performed screening for porcine GAPDH using specific primers (Table 2).

### 2.7. Determination of the PERV Copy Number

Droplet digital PCR (ddPCR) was performed according to the manufacturer’s instructions (Bio-Rad, Hercules, CA, USA, [http://www.bio-rad.com/de-de/applications-technologies/droplet-digital-pcr-ddpcr-technology?ID=MDV31M4VY]) using a QX200 droplet generator and a QX100 droplet reader (Bio-Rad). Purified genomic DNA from cultured cells (50 ng genomic DNA) was digested with MseI (New England Biolabs, Ipswich, MA, USA) (20U) at 37 °C for 1 h. The ddPCR mix consisted of 10 μL 2× ddPCR Master mix, 1.8 μL of each 10 µM target primers (Table 2), 0.5 µL of each 10 µM probes (FAM/HEX) (Table 2). The DNA digest had a concentration of 2.5 ng/µL and 2 µL corresponding to 5 ng digested DNA and water was added to a total volume of 20 μL. The following cycling conditions were used: 10 min initial enzyme activation at 95 °C, 30 s denaturation at 94 °C, 1min annealing and extension at 60 °C (40 cycles) and final 10 min enzyme deactivation at 98 °C using a Master cycler ProS (Eppendorf). The temperature ramp rate was 2 °C per second.

### 2.8. Transmission Electron Microscopy

PERV-producing 293 T cells were fixed with 2.5% glutaraldehyde in 50 mM HEPES, pH 7.2. The cells were harvested by scraping, pelleted at 2.000 g for 5 min at 4 °C, and washed twice with HEPES. After washing, the cells were block-embedded by mixing equal amounts of pelleted cells and low-melting-agarose (3%). Agarose-embedded cells were cut into small pieces (<1 mm), and postfixed with osmium tetroxide (1% in double distilled H_2_O for 1 h), tannic acid (0.1% in 50 mM HEPES for 30 min), and uranyl acetate (2% in ddH_2_O for 2 h). The agarose-embedded cells were dehydrated in a graduated ethanol series and finally embedded in Epon resin. Thin sections (60–70 nm) were cut on a Leica-Ultracut ultramicrotome, mounted on naked 300 mesh copper grids, and counterstained with uranyl acetate (2% in ddH_2_O for 20 min), followed by lead citrate (Reynolds’ solution for 3 min). Ultrathin sections were stabilized with a thin layer of carbon evaporation and examined using a JEM-2100 transmission electron microscope (JEOL) at 200 kV. Images were recorded using a Veleta CCD camera (EMSIS).2.8. HEV ELISA.

To test for antibodies against HEV, the PrioCHECK HEV Antibody ELISA Kit, porcine (Applied Biosystems) was used. Porcine plasma, the controls, which are included in the kit, and sera from previous positive tested pigs are analysed. Recombinant hepatitis E virus antigens encoded by the open reading frames (ORF) ORF 2 and ORF 3 of the genotypes 1 and 3 were coated on the ELISA plate. After incubation of the samples with the antigen (60 min, 37 °C) a washing step followed. The anti–HEV antibodies present in the sample were incubated with the peroxidase (POD)-labeled anti-pig IgG conjugate (30 min, 37 °C) and the unbound conjugate was washed away. 3,3′,5,5′-tetramethylbenzidine (TMB) substrate was used for color development, which was measured at a wavelength of 450 nm and a reference filter at 620 nm.

## 3. Results

### 3.1. Brief History of Göttingen Minipgs

To date, the Göttingen Minipig (GöMP) is among the smallest commercially used pig breeds in the world, and it has been under a well-documented breeding scheme since its foundation in the 1960s. Its beginnings date back to 1949, when first efforts to establish a breeding program for one of its ancestors, the Minnesota Minipig, were undertaken by Prof. Winters and his co-workers at the Hormel Institute in Austin, Minnesota [34]. With the goal to attain a pig of limited size for laboratory research, they collected and crossed individuals from several small-sized, wild and feral breeds from the U.S., among them feral hogs from Alabama, Piney-Woods-Hogs from Louisiana, a boar, probably of the genus *Sus scrofa vittatus*, from the Island of Santa Catalina, California, and Ras-n-Lansa pigs from Guam. The resulting pigs were generally of small stature, but highly variable in body size and coat color.

Prof. Fritz Haring and his co-workers from the Institute of Animal Breeding and Genetics of the Georg-August-University Göttingen, Germany, recognized the demand for a similar animal model in Europe, and were able to obtain five individuals from Minnesota in 1960. They crossed them with Vietnamese Potbellied Pigs from zoological gardens in West and East Germany. In 1965, another four Vietnamese Potbellied Pigs were acquired. The resulting crosses were small, of short stature, mostly obese and of diverse coat color. German Landrace sires were introduced by artificial insemination to introgress dominant white coat color. Several generations of reciprocal mating with minipigs and strong selection for small body size eventually resulted in a uniformly white minipig, when the herdbook was closed in 1969 [35].

The GöMP is nowadays bred in five genetically isolated sub-populations, four of them run by licensees, in Germany, Denmark (two colonies), USA and Japan. This strategy is beneficial from a market perspective, since location of production units close to the relevant markets reduces animal transports, which is also highly beneficial from an animal welfare point of view. The units remain isolated, since exchange of animals would harbor the risk of transferring diseases or hidden genetic disorders. A recent study has shown that albeit being isolated for decades, all stocks are genetically still highly similar due to a common breeding effort [36].

### 3.2. Screening for PERV-C

**Figure 3:**
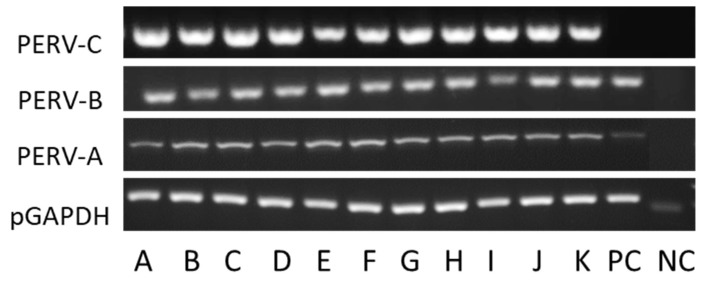
Detection of PERV proviruses in 11 GöMPs using a PCR method. PC, PK15 cells: NC, negative control, water.

All GöMP from Göttingen were found positive for PERV-A and PERV-B (Figure 3). When the animals were screened for PERV-C a PCR method and a real-time PCR were used (Table 1). All 11 animals were found positive for PERV-C with both methods (Table 1) (Figure 3).

### 3.3. Screening for PERV-A/C

**Figure 4:**
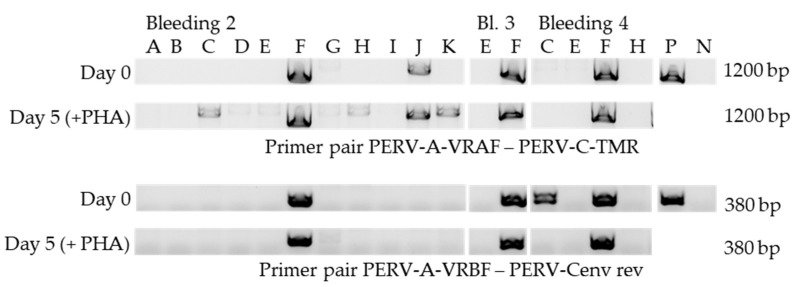
Detection of PERV-A/C proviruses in GöMP from the Göttingen University. PBMCs from three consecutive bleedings (bleedings 2, 3 and 4) were isolated and tested at day 0 and after 5 days of cultivation with PHA. Two primer pairs were used resulting in overlapping amplicons (Figure 2). 11 pigs (A–K) were analyzed, as well as a PERV-A/C plasmid PERV-A 14/220 ([37], AY570980) (P) and as negative control (N) water.

Using specific primers (Table 2), the animals were screened for the prevalence of PERV-A/C in the genome of PBMCs. For this, DNA was isolated from PBMCs at day 0 and after cultivation of the PBMCs in culture medium in the presence or absence of PHA-L after 5 days. This experiment was repeated in some cases (pig E and F) three times and in the case of pigs C and H two times. This means three consecutive bleedings of pigs E and F were cultured with PHA-L and analyzed by PCR for PERV-A/C prevalence. Using the primer pair PERV-A-VRBF/PERV-C-TMR, a PERV-A/C amplicon (1200 bp) was found in PBMCs from pig F and J already on day 0, in PBMCs from animals C and K only after 5 days stimulation (Figure 4). Using a reverse primer located inside the sequence amplified by the above mentioned primers, a shorter amplicon was obtained confirming the presence of PERV-A/C in PBMCs from animal F. Interestingly, using these primers in PBMCs from pig C, a specific sequence was amplified, whereas the longer sequence with the other primer was not found, indicating that the primer binding site for the primer PERV-C TMR is missing or mutated and that the PERV-A/C in animal C has a different recombination breakpoint compared with that of animal F. PERV-A/C had not been found in GöMP from Ellegaard [6].

### 3.4. Copy Number of PERV in the Genome

**Figure 5:**
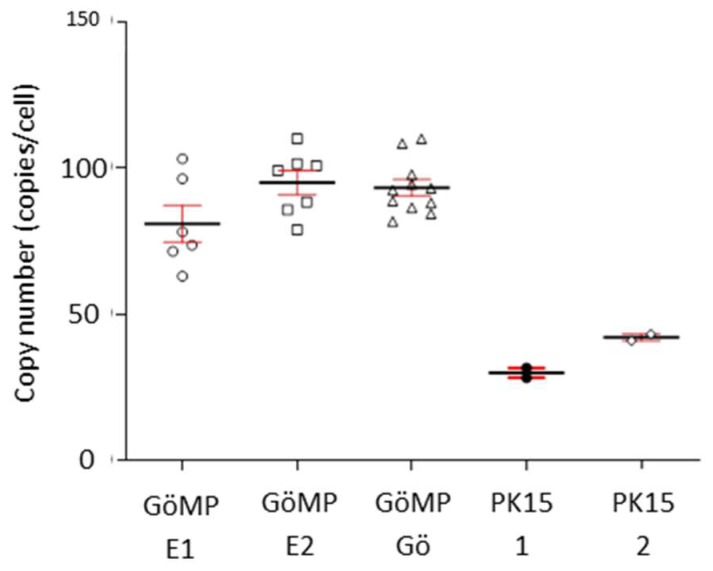
Determination of the copy number of PERV in the genome of Göttingen Minipigs (GöMP) from Ellegaard (E) and from the University of Göttingen (Gö) using GAPDH as reference. In addition, PK15 cells were tested. Results under number one (1) were published in Fiebig et al. [10], number two (2) represent data obtained in this study together with the testing of GöMP from Göttingen.

**Figure 6:**
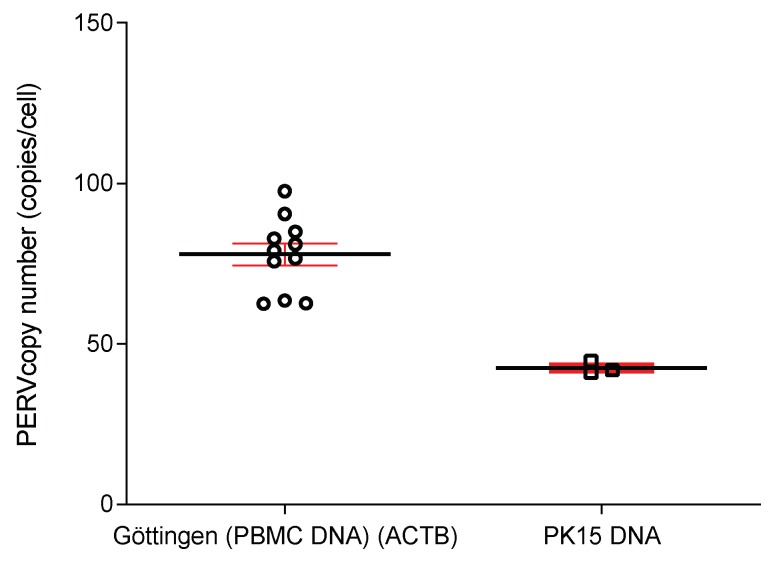
Determination of the PERV copy number in PBMCs from GöMP from Göttingen and in PK15 cells using ACTB as reference.

Using ddPCR the PERV copy number was determined. The copy number measured in the PBMCs from blood of the GöMP from Göttingen was 93+/–9 per cell, the copy number in GöMP from Ellegaard was 95+/–10, in both cases using porcine GAPDH (pGAPDH) as reference (Figure 5). These data were higher when compared with the copy number in GöMP from Ellegaard reported previously (82+/–12) [10]. As a control the copy number of PERV in PK-15 was determined: 42+/–1 copies. In a previous publication a copy number of 30+/–1.6 was found, also using pGAPDH as reference [10]. As previously shown, the number of actin genes (ACTB) and GAPDH genes was not always two per cell as theoretically expected [10], and so we repeated the analysis using ACTB as reference. Using this setting, the mean PERV copy number in PBMCs from the GöMP from Göttingen was 77.91, and in PK15 cells 42.53 (Figure 6). The ratio of the PERV copy number in PK15 cells estimated using GAPDH as reference gene (42 copies), to the copy number determined using ACTB (30 copies), is 1.4, suggesting that in these cells approxiomately two GAPDH genes and three ACTB genes are present. The situation is similar in the GöMP from Göttingen, the ratio of the copy number estimated using GAPDH as reference gene (93 copies), to the copy number determined using ACTB (72 copies), is 1.3. Therefore, the copy number of PERV depends on the copy numbers of the reference genes.

### 3.5. Expression of PERV

**Figure 7:**
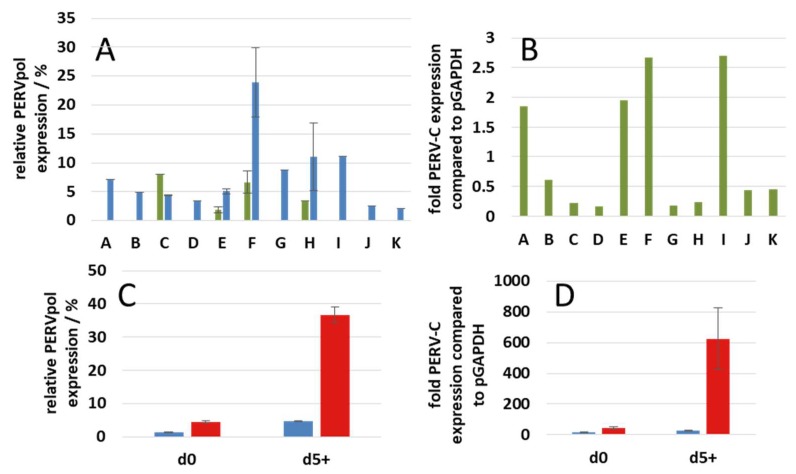
Relative expression of all PERVs using primers specific for pol (**A**,**C**) and primers specific for PERV-C env (**B**,**D**). (**A**), Expression of PERVpol at day zero (green column) and day 5 after PHA stimulation (blue columns) relative to the expression in PK15 cells. Eleven pigs (A, B, C, D, E, F, G H, I, J and K) were tested. PBMCs from pigs A, B, D, G, I, J and K were tested once, the PBMCs from pigs C and H were tested twice (two bleedings with independent PHA-L stimulations), the PBMCs from pigs E and F six times (three different bleedings with independent PHA-L stimulations). In the case of pigs A, B, D, G, I, J, and K no measurement at day 0 were performed. (**B**), expression of PERV-C at day 0 compared to the expression of GAPDH, (**C**), relative expression of PERVpol compared with the expression of PK15 cells on day 0 (d0) and day 5 (d5) after stimulation with PHA (PBMCs from animal E, blue column, PBMCs from animal F, red column), (**D**), expression of PERV-C on day 0 (d0) and day 5 (d5) after stimulation with PHA-L compared to the expression of GAPDH (PBMCs from pig E, blue column, PBMCs from pig F, red column). All error bars indicate s.e.m.

To study the expression of PERV, a RT-qPCR was performed using primers specific for the pol region. This PCR measures the full-length mRNA of all PERVs. The full-length mRNA encodes the Gag and Pol proteins. All animals expressed this mRNA in their freshly isolated PBMCs, animal E only at a very low rate (Figure 7A). After stimulation with 2.4 µg/mL PHA-L the expression was high in PBMCs from pig F, lower in PBMCs from pigs G, H, and I and low in the PBMCs from the other pigs (Figure 7A).

Using specific primers, the expression of PERV-C was analyzed. The highest expression was found in PBMCs from animals F and H, slightly lower in PBMCs from pigs A and E, and low in the other pigs (Figure 7B). The expression of PERV-C is of importance, because PERV-C is required in order to generate PERV-A/C. High expression of PERV-C was found in PBMCs from the same animals showing a high expression of full-length mRNA (pigs F and I). The other animals showed a low expression.

As shown previously, the expression of PERV increases after stimulation of PBMCs from pigs of different strains with PHA [6,24,25]. These findings were confirmed here. Both the expression of the full-length mRNA detected by PERV pol primers (Figure 7C) and the expression of PERV-C (Figure 7D) increased in PBMCs from pig E and pig F. The increase in PBMCs from pig F is much higher when compared with that from pig E. The expression of PERV-A/C increased also significantly (not shown). The use of higher concentrations of PHA-L (9.6 µg/mL) did not results in higher PERV expression rated.

To screen for spliced mRNA, which is encoding for the Env proteins, specific primers were used as shown in Figure 2. Spliced mRNA was not found in freshly isolated PBMCs, but in cultured and stimulated PBMCs after 5 days with exception of pig A (Table 3).

### 3.6. Release of Human-Tropic PERV

To study the release of infectious virus particles, mitogen-stimulated PBMCs from all GöMP were co-cultured with human 293 cells, which are highly sensitive for PERV infection (Figure 1). Only PBMCs from a single animal, pig F, released particles able to infect 293 T cells (Table 1). The virus was found released in three different experiments, e.g., three consecutive bleedings obtained from the pig F with independent co-cultivations with 293 T cells. The virus, designated F-PERV, was able to infect uninfected 293 cells and was identified as PERV-A/C by PCR (Figure 4).

**Figure 8:**
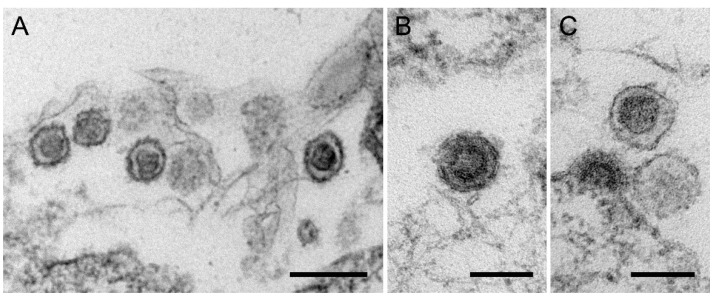
Electronmicroscopy of F-PERV particles produced in 293 cells. (**A**), a group of mature viruses, (**B**), a maturing virus, (**C**), a budding and a mature virus. The bar corresponds to 200 nm (**A**) and 100 nm (**B**,**C**).

Electron microscopy analysis of 293 cells in the 14th passage after co-cultivation with PBMCs from pig F showed typical type C viruses. Mature, maturing (Figure 8A,B) and budding viruses (Figure 8C) were found.

**Figure 9:**
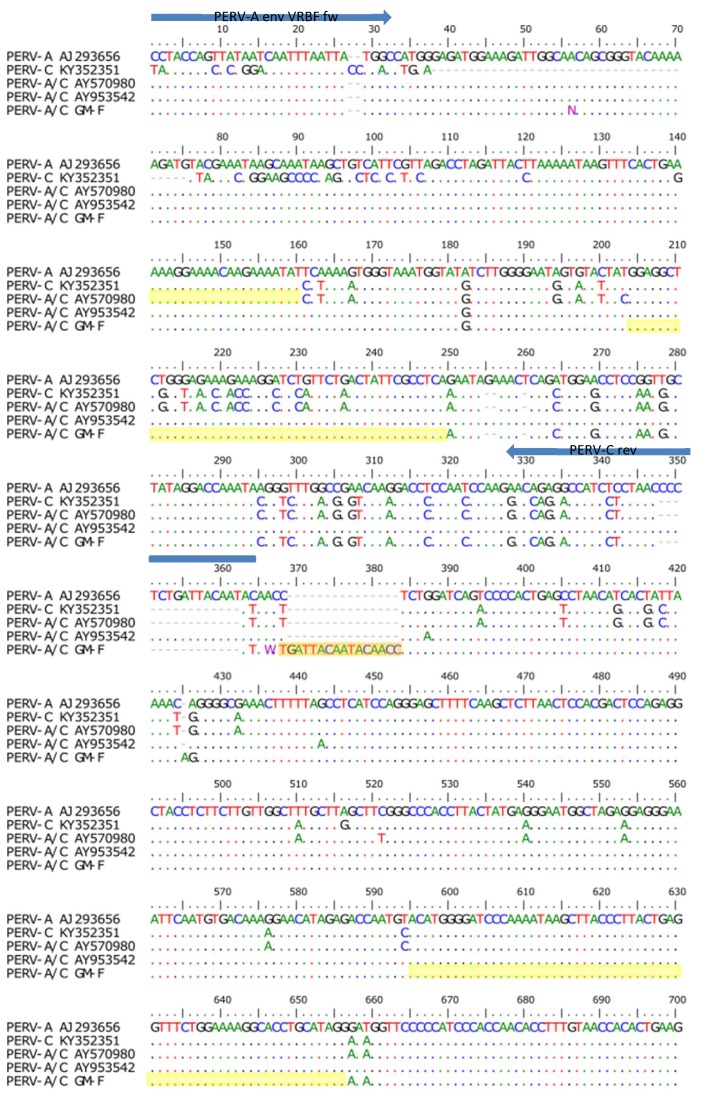

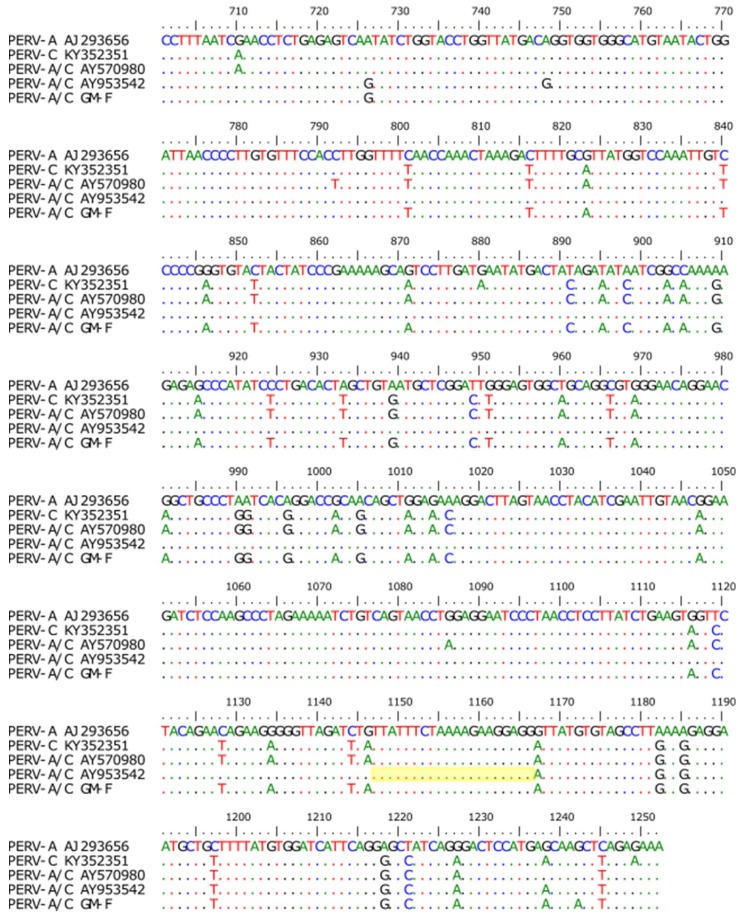
Sequence alignment of PERV-A, PERV-C, and two PERV-A/C, the PERV-A 14/220 described by Bartosch et al. [37] (AY570980) and the PERV-A/C 5^0^ analyzed in our laboratory (AY953542) [9,23,38], as well as the virus isolated from GöMP F (GM-F). The primer PERV-A VRBF (blue line) and the primer PERV-C-TMR (not shown, ranging from nt 1288–1263) (Table 2) were used for amplification and sequencing of the PERV-A/C env sequence. The primer PERV-C rev (blue line) was also used for the detection of PERV-A/C (Figure 4). The sequences marked yellow represent the potential regions of recombination, the orange bar indicate an insertion. N, any nucleotide; M, A or C, W, A or T.

Sequencing of the amplicon obtained in the PERV-A/C specific PCR confimed that it is a PERV-A/C (Figure 9). The region of recombination between PERV-A and PERV-C was identified between nt 205 and 249 of the sequenced region which is between nt 721 and 811 of the env gene and a second breakpoint was found between 596 and 657. Therefore, the organisation of the sequence is PERV-A-C-insert-A-C.

### 3.7. Screening for PCMV, HEV, PLHV, PCV2 and PCV3

Using specific primers and testing the blood of the animals or the isolated and cultivated PBMCs for PCMV, HEV, PLHV-1, PLHV-2, PLHV-3, PCV2 and PCV3, all samples were found negative (Table 1). Based on these results differences between the GöMP at Ellegaard and at the University of Göttingen were identified (Table 4).

## 4. Discussion

This is the first extensive virological characterization of GöMPs breed isolated at the University of Göttingen in comparison with the previously characterized GöMPs breed at Ellegaard. It is also the first report showing a comprehensive strategy testing for 11 different xenotransplantation-relevant porcine viruses in the donor pigs, including PERV-A, PERV-B, PERV-C and PERV-A/C as well as analyzing transmission of human-tropic PERVs to human cells (Figure 1). This strategy is based on blood of donor pigs.

Among the GöMP produced at Ellegaard HEV, PCMV, PCV2 and PLHV were found in a very small number of animals [11,12] (Table 4) and it was shown that HEV was transmitted via the placenta [11]. It is interesting that the GöMP produced in Göttingen are free of PCMV, HEV, PLHV-1, PLHV-2, PLHV-3, and PCV3. Either the infections with PCMV and HEV viruses happened at Ellegaard, or due to the small number of animals investigated in Göttingen, PCMV-positive and HEV-positive animals were missed. Infections could have happened during transport in containers used previously for other pigs, or direct in the facility or by contaminated personal or material.

The data show that all GöMP in Göttingen contained PERV-A, PERV-B and PERV-C in their genome. The presence of PERV-C is a prerequisite for recombinations with PERV-A, resulting in high titer PERV-A/C recombinants [8,9]. Since PERV-A/C proviruses were already found in unstimulated PBMCs, it is highly likely that these viruses can also be found in the living pig. In contrast, recombinant PERV-A/Cs were not found in PBMCs from five Ellegaard pigs analyzed previously [6].

One of the main finding of this study is that PBMCs of only one from 11 GöMP from Göttingen released a human-tropic PERV. This virus was identified as PERV-A/C (Figure 4 and Figure 9). In parallel, 39 other pigs were analyzed using the same strategy including five Black forest Minipigs, five Aachen Minipigs, 18 German Landrace and Large White cross-breeds and 5 others, none of them released a human-tropic PERV able to infect 293 cells. Therefore, only one from 50 pigs released human-tropic PERV [38]. In contrast to what has been published before [9,37], in the sequence of the recombinant F-PERV there are two recombination points and an insertion of 15 nucleotides (Figure 9).

To evaluate the potential risk posed by PERVs is quite difficult. PERV-A, PERV-B and PERV-C are integrated in the genome of all pigs, including the GöMP (Figure 3). PERV-A and PERV-B are able to infect human cells, mainly human 293 cells lacking intracellular restriction factors [27]. In contrast to this cell line, infection of human primary cells was achieved only with PERV passaged on human cells [9,23,39]. Passaging the virus had resulted in additional repeats in the LTR and an increase of the number of transcription factor binding sites and consequently in an increased replication rate [9]. At present, infection of 293 cells is thought to be the best assay to study the release of human-tropic PERVs by pig cells [40]. This assay is widely accepted by regulatory agencies, but it is a complex, expensive and time-consuming assay, especially if required at numerous stages in the process of preparation of a transplant. In order to replace this assay, we suggested alternative assays based on molecular biological methods [41]. One proposed assay is based on the detection of spliced PERV-specific mRNA in mitogen-stimulated pig PBMCs, another is based on screening for PERV-A/C, since there are indications that only PERV-A/C are released from mitogen-activated PBMCs and infect human cells.

Here we performed for the first time all assays in parallel. Using a PCR method, spliced mRNA was only detected in cultured pig PBMCs regardless whether they were stimulated with PHA or not (with exception of pig A), but not in freshly isolated PBMCs (Table 3). The culture in medium with fetal calf serum (FCS) is certainly also associated with an immune stimulation of the pig PBMCs. These preliminary data suggest that the detection of spliced mRNA is not a valuable marker for virus release, but the detection of PERV-A/C is a good correlate to the release of infectious virus. Although only one animal released a human-tropic PERV, the fact that it is a PERV-A/C supports previous findings that only recombinant PERV-A/C are released from mitogen-stimulated PBMCs and that these viruses are able to infect human 293 cells [25]. The situation may be different in the case of other cells transplanted as part of solid organs or islet cells for the treatment of diabetes. The virus release from stimulated PBMCs may represent a kind of worst-case scenario, which may not happen with other cells.

In order to release virus, the cells should produce high amounts of mRNA required for protein production and viral RNA to be packed into the virions. It is important to note, that the animal F, which released a human-tropic PERV-A/C, had the highest expression of full- length mRNA and together with animals A and I the highest expression PERV-C mRNA (Figure 6).

As mentioned above, only one of 11 GöMP from Göttingen released infectious PERV-A/C particles. Higher expression of PERV and a higher prevalence of PERV-A/C have been linked with poor health and disease in pigs in commercial swine operations [42]. However, the GöMP in Göttingen are produced under highly hygienic conditions and therefore it is likely that the differences in PERV expression and the release of PERV-A/C in one animal are not associated with infections, but with a genetic trait. There is also no correlation with age (Table 1).

Stimulation of PBMCs from GöMP from Göttingen with a T cell mitogen, PHA-L, increased the expression of PERV (Figure 7C,D). This confirms results with stimulated and unstimulated PBMCs from GöMP from Ellegaard where an increase of PERVpol and PERV-C expression had been observed on day 3 and day 5 compared with day 0 [6]. This also agrees with previous data showing that the amount of released virus increased in the supernatant of stimulated PBMCs after 5 days [24]. Until now, in all preclinical trials transplanting pig islet cells, kidneys and hearts to non-human primates no PERV transmission was observed [43]. However, it has to be considered, that the PERV receptor in non-human primates is mutated and therefore not fully functional. No transmission of PERV was also observed in the first clinical trials, transplanting pig islet cells into diabetic patients in New Zealand and Argentina under regulatory oversight [44,45]. At present there are no further experimental opportunities to evaluate the potential risk posed by PERV until we move to the clinic [46]. On the other hand, there were several attempts to reduce the risk of PERV transmission by reduction of the PERV expression using PERV-specific siRNA in vitro and in vivo [47,48,49,50,51,52], by PERV-specific vaccines [53,54,55,56], antiviral drugs (for review see [57]) and by genome editing. Whereas first attempts to inactivate multiple PERV sequences in the pig genome using zinc finger nuclease failed [58], inactivation by CRISPR/Cas9 seemed successful [21] and using this technology, healthy piglets with inactivated PERVs were generated [59].

Although until now no transmission of PERV to the recipients in all preclinical and clinical trials was observed (for review see [60]), it is still unclear at present, whether genome edited animals should be used for clinical xenotransplantation or whether PERV-C negative pigs will be safe enough [61,62,63]. Furthermore, transplanting islet cells from GöMP produced at Ellegaard into cynomolgus monkey was also not associated with transmission of PERV or other porcine viruses [16]. A clinical study of transplantation of islet cell from GöMP is planned in near future in Germany.

## 5. Conclusions

Using a new comprehensive strategy, a population of GöMP, which was bred isolated at the University of Göttingen, was analyzed for the presence of 11 xenotransplantation-relevant porcine viruses including PERV. This strategy tested GöMP for viruses by PCR, RT-PCR and immunological methods. Furthermore, combining co-cultivation assays with susceptible human target cells and molecular biological methods the expression and transmission of PERV was investigated. This strategy was very effective in order to analyze the risk posed by PERV. The results show that the GöMP from the Göttingen University are less contaminated with porcine viruses and therefore also suited for use in clinical xenotransplantation after removal of the remaining viruses. The differences between GöMP at the University of Göttingen and Ellegaard may be explained by selection of negative animals and/or de novo infection. On the other hand, the GöMPs from Göttingen showed a higher prevalence of PERV-A/C in their PBMCs and in one case release of an infectious human-tropic PERV-A/C was observed.

## Figures and Tables

**Table 1:** Overview of the screening of Göttingen Minipigs from the University Göttingen (blood was taken on 8 February 2019 and 1 March 2019, and for some animals again on 14 May 2019 and 28 August 2019.

**ID**	Sex	Date of Birth	Remarks	Virus										
				PCMV	PERV			PCV2	PCV3	PLHV-1	PLHV-2	PLHV-3	HEV	
				Real-Time PCR	PERV-C Real-Time PCR	PERV-C EnvC2 PCR	PERVpol PCR	PCR	Real-Time PCR	PCR	PCR	PCR	ELISA	Real-Time PCR
A	M	8 June 2014	Father (sire)	-	+	+	+	-	-	-	-	-	-	-
B	F	14 May 2015		-	+	+	+	-	-	-	-	-	-	-
C	F	17 October 2015	Mother (dam)	-	+	+	+	-	-	-	-	-	-	-
D	F	17 October 2015		-	+	+	+	-	-	-	-	-	-	-
E	F	29 May 2016		-	+	+	+	-	-	-	-	-	-	-
F	F	14 October 2026		-	+	+	+	-	-	-	-	-	-	-
G	F	7 July 2017	Daughter (female offspring)	-	+	+	+	-	-	-	-	-	-	-
H	M	25 October 2017		-	+	+	+	-	-	-	-	-	-	-
I	M	14. January 2018		-	+	+	+	-	-	-	-	-	-	-
J	F	31 May 2018		-	+	+	+	-	-	-	-	-	-	-
K	F	23 June2018		-	+	+	+	-	-	-	-	-	-	-

+ means positive detection of the virus, - means absence of virus.

**Table 2:** Primers and probes used for virus screening.

Primers Used	Sequence 5′-3′	Nucleotide Position	Accession Number	Reference
**Primers used for PCR**				
PLHV-1,-2 (747) fw PLHV-1,-2 (747) rev	CAYGGTAGTATTTATTCAGACA GATATCCTGGTACATTGGAAAG	21,146–21,167 21,488–21,467	AY170317.1	Ehlers B., 2002 [28]
PLHV-3 (905) fw PLHV-3 (905) rev	ACAAGAGCCTTAGGGTTCCAAACT GTGTCCAGTGTTGTAATGGATGCC	13,472–13,495 13,727–13,704	AY170316.1	Chmielewicz et al., 2003 [29]
PCV2 fw (F66) PCV2 rev (B67)	GGTTTGTAGCCTCAGCCAAAGC GCACCTTCGGATATACTGTCAAGG	567–546 152–175	KT868491.1	Mankertz et al., 2000 [30]
PERV env C.2 fw PERV env C.2 rev	GATTAGAACTGGAAGCCCCAAGTGCTCT TCTGATCCAGAAGTTATGTTAGAGGATGGT	9362–9389 9649–9620	AM229312	Dieckhoff et al., 2009 [26]
**Primers and probes used to detect PERV-A/C**				
PERV-A-VRBF PERV-C-TMR PERV-C rev	CCTACCAGTTATAATCAATTTAATTATGGC C TCAAACCACCCTTGAGTAGTTTCC TATGTTAGAGGATGGTCCTGGTC			Wood et al., 2004 [7] Dieckhoff et al., 2009 [26]
**Primers and probes used for real-time PCR**				
PCV3 fw PCV3 rev PCV3 probe	AGTGCTCCCCATTGAACG ACACAGCCGTTACTTCAC FAM-ACCCCATGG-Zen-CTCAACACATATGACC-BHQ	1427–1444 1561–1544 1473–1449	KT869077	Palinski et al., 2016 [31]
pGAPDH fw pGAPDH rev pGAPDH probe	GATCGAGTTGGGGCTGTGACT ACATGGCCTCCAAGGAGTAAGAHEX-CCACCAACCCCAGCAAGAGCACGC-BHQ	1083–1104 1188–1168 1114–1137	NM_001206359.1	Duvigneau et al., 2005 [32]
HEV fw HEV rev HEV probe	GGTGGTTTCTGGGGTGAC AGGGGTTGGTTGGATGAA FAM-TGATTCTCAGCCCTTCGC-BHQ	5261–5278 5330–5313 5284–5301	M73218.	Jothikumar et al., 2006 [17]
PCMV fw PCMV rev PCMV probe	ACTTCGTCGCAGCTCATCTGA GTTCTGGGATTCCGAGGTTG FAM-CAGGGCGGCGGTCGAGCTC-BHQ			Mueller et al., 2002 [33]
PERV pol fw PERV pol rev PERV pol probe	CGACTGCCCCAAGGGTTCAA TCTCTCCTGCAAATCTGGGCC FAM-CACGTACTGGAGGAGGGTCACCTG-BHQ	3568–3587 3803–3783 3655–3678	HM159246	Yang et al., 2015 [21]
**Primers used to detect spliced mRNA**				
P1 P2 P3	TGCTGTTTGCATCAAGACCGC ACAGACACTCAGAACAGAGAC ATGGAGGCGAAGCTTAAGGGGA			Karlas et al. [23]
**Primers and probes used for dd PCR**				
pGAPDH fw pGAPDH rev pGAPDH probe	TTCACTCCGACCTTCACCA CCGCGATCTAATGTTCTCTTTC HEX-CAGCCGCGTCCCTGAGACAC-BHQ	3951–3970 4022–4001 3991–3972 bp	396823	Yang et al., 2015 [21]

**Table 3:** Expression of PERV in stimulated and unstimulated PBMCs from GöMP.

ID	PERV Expression							Release of Virus ^3^
	Non-Stimulated, Non-Cultured		Non-Stimulated, Cultured		Stimulated, Cultured			
	FL ^1^	S ^2^	FL ^1^	S ^2^	FL ^1^		S ^2^	
A	+	-	+	-	+		-	-
B	+	-	+	+	+		+	-
C	+	-	+	+	-+		+	-
D	+	-	+	+	-+		+	-
E	+	-	+	+	+		+	-
F	+	-	+	+	+		+	+
G	+	-	+	+	+		+	-
H	+	-	+	+	+		+	-
I	+	-	+	+	+		+	-
J	+	-	-+	+	+		+	-
K	+	-	+	+	+		+	-

^1^ FL, full length mRNA; ^2^ S, spliced mRNA; ^3^ human-tropic virus infecting 293 T cells.

**Table 4:** Comparison of GöMP at Ellegaard and the University of Göttingen.

Virus	Ellegaard ^1^	University Göttingen
PERV-A, PERV-B	40/40 (100%)	11/11 (100%)
PERV-C	28/28 (100%)	11/11 (100%)
HEV	9/40 (22.5%)	0/11 (0%)
PCMV	10/22 (45%)	0/11 (0%)
PLHV-1	1/10 (10%)	0/11 (0%)
PLHV-2	n.t.	0/11 (0%)
PLHV-3	n.t.	0/11 (0%)
PCV2	3/21 (14%)	2/11 (18%)
PCV3	0/10 (0%)	0/10 (0%)

^1^ Data from [6,11,12,16,19].
